# Radiolysis of myoglobin concentrated gels by protons: specific changes in secondary structure and production of carbon monoxide

**DOI:** 10.1038/s41598-024-58378-z

**Published:** 2024-04-14

**Authors:** Nicolas Ludwig, Catherine Galindo, Clea Witjaksono, Antoine Danvin, Philippe Peaupardin, Dominique Muller, Tamon Kusumoto, Satoshi Kodaira, Rémi Barillon, Quentin Raffy

**Affiliations:** 1 IPHC, UMR 7178, Université de Strasbourg and CNRS, 23 rue du Loess, F-67037 Strasbourg, France; 2 Aerial, Parc D’innovation, 250 Rue Laurent Fries, F-67400 Illkirch, France; 3 Institut de Chimie, UMR 7177, Université de Strasbourg and CNRS, 4 rue Blaise Pascal, F-67070 Strasbourg, France; 4 ICube, UMR7357, Université de Strasbourg and CNRS, 23 rue du Loess, F-67037 Strasbourg, France; 5 National Institutes for Quantum and Radiological Science and Technology (QST), 4-9-1 Anagawa, Inage-ku, Chiba, 263-8555 Japan

**Keywords:** Radiotherapy, Proteins, Physical chemistry

## Abstract

While particle therapy has been used for decades for cancer treatment, there is still a lack of information on the molecular mechanisms of biomolecules radiolysis by accelerated ions. Here, we examine the effects of accelerated protons on highly concentrated native myoglobin, by means of Fourier transform infrared and UV–Visible spectroscopies. Upon irradiation, the secondary structure of the protein is drastically modified, from mostly alpha helices conformation to mostly beta elements at highest fluence. These changes are accompanied by significant production of carbon monoxide, which was shown to come from heme degradation under irradiation. The radiolytic yields of formation of denatured protein, carbon monoxide, and of heme degradation were determined, and found very close to each other: G_+denatured Mb_ ≈ G_+CO_ ≈ G_-heme_ = 1.6 × 10^–8^ ± 0.1 × 10^–8^ mol/J = 0.16 ± 0.01 species/100 eV. The denaturation of the protein to a beta structure and the production of carbon monoxide under ion irradiation are phenomena that may play an important role in the biological effects of ionizing radiation.

## Introduction

Particle therapy, or hadron therapy, is a form of cancer radiotherapy used to treat tumors that are generally inoperable, positioned in a sensitive region (i.e. eye or brain) or resistant to traditional radiotherapy treatments. It is based on irradiations with accelerated ions, mainly carbon ions and protons^1^. An understanding of the biological effects of energetic protons at various scales, from tissue to cellular and molecular levels, is thus critical to optimize proton radiotherapy. When entering a cell, energetic protons interact with biological matter (proteins, DNA, lipids etc.) either directly or indirectly, through radiolysis of water, which makes up about 70% of the cell in mass. Water radiolysis produces several reactive species (e^−^_aq_, H^•^, HO^•^, H_2_O_2_), among which hydroxyl radical HO^•^ is the most potent one. HO^•^ reacts very quickly with nearby molecules, resulting in bond-breaking, hydroxylation or oxidation of the molecules^2^. As its damages can lead to cell death, DNA is a critical target of hydroxyl radicals in the cell and several studies have focused on the effects of irradiations on this biomolecule. Radiation-induced DNA damages consist in base fragmentations, deletions, single or double strand breaks, and cluster damages, which are particularly difficult to repair^3–6^. On the other hand, proteins are the most abundant component of the cell after water, accounting for up to 20% in mass. They will thus statistically undergo the most damage upon irradiation. They are involved in virtually all cellular functions, from the transport of molecules to the catalysis of metabolic reactions, including immune protection, transmission of nerve impulses, DNA repair and many others. It is therefore critical to better understand how radiations will affect them. Protein degradation as a result of gamma or X-ray irradiation leads to fragmentation, cross-linking, aggregation or denaturation^7–9^. Now, a denatured protein can display a very different activity profile than the protein in its native form, usually losing its biological function, which is closely related to its secondary structure^10^. To date, studies related to protein irradiation by ions are scarce^11–14^, especially under native conditions, and the question remains as to the impact of accelerated ions on their secondary structure and more generally on molecular modifications. While therapy is performed with high-energy protons, their initial energy is chosen so that their trajectory ends with the Bragg peak in the tumor, after losing most of their energy in the surrounding tissues. Therefore, in the tumor, their remaining energy is quite low, of a few MeV. In this work, we studied at the molecular level the effect of 2 MeV protons on native hydrated myoglobin, focusing on the changes of its secondary structure and transformations of the heme.

Myoglobin is found in abundance in myocyte cells of heart and skeletal muscles of mammals^15^, and belongs to the large class of heme-proteins. It exhibits a globular shape in solution, and has been extensively studied for many years in relation to protein structure^16,17^. Horse hearth myoglobin, used in this study, consists in 8 α-helices, connected by short non-helical regions, mainly beta turns and random coils, without any disulphide bond (RCSB entry 1WLA^18^). The strong absorption of the protein heme allows following the redox state and axial ligands of the central iron by UV–Visible spectroscopy. Its radiolysis has been described in diluted solutions with gamma rays or electrons in several studies^19–23^. In aerated medium, reaction with water radiolysis species leads to formation of ferrimyoglobin-peroxide and reduction of the Iron(III) heme to deoxymyoglobin. On the protein itself, it can lead to dimerization, aggregation, or peptide chain scission.

This, together with its well-described secondary structure, makes it a prime target for this study, as a model heme protein.

## Results

The effects of low-energy proton irradiation on met-Myoglobin characteristics were investigated in aqueous media, at high concentration. To do so, native protein was irradiated, in samples thin enough to allow FTIR and UV–Visible analyses, and for 2 MeV protons to pass through. At this energy, the average penetration depth of protons into water is 77 µm. The concentration of the protein was chosen to be representative of the average global protein content in living cells, i.e. around 20% w:w. In order to avoid flowing of a solution in a vertical position, and maintain a constant thickness of the sample throughout irradiation, self-consistent deuterated protein hydrogels were prepared. Glutaraldehyde was used as cross-linking agent, at a 1:3.3 protein:glutaraldehyde molar ratio. These gels were prepared with thicknesses of 10–30 µm, controlled by infrared and UV–Vis spectroscopies, and were stable over several hours, especially in terms of hydration. Protein gels were prepared in deuterated water, to allow observation of the protein's Amide I band by infrared spectroscopy. The H_2_O bending band is located at 1650 cm^−1^, and would therefore be superimposed on the amide I band, while that of D_2_O is found at 1210 cm^−1^.

### Exploitation of the FTIR amide I band

The Amide I vibration of the polypeptide backbone contains data allowing the determination of the secondary structure of the macromolecule^24^. Proportions in alpha helices, beta and random structures of the protein were determined after calculation of the smoothed second derivative of the Amide I band and its deconvolution, based on a procedure described by Yang et al.^25^.

For the myoglobin gel, seven peaks were identified, at 1649 cm^−1^ (alpha helices), 1639 cm^−1^ (random coils), 1662, 1670, 1680, 1694 cm^−1^ (beta turns) and 1628 cm^−1^ (beta sheets). Using the areas of these peaks, proportions of 70 ± 5% alpha, 20.5% ± 1% beta and 9.3 ± 5% random were obtained for the native protein, in agreement with crystal structure of the protein^18^. For the radiolysis experiments, infrared spectra were recorded after each irradiation dose. Irradiation of myoglobin gel by 2 MeV protons resulted in a slight decrease in intensity of the Amide I band, together with its enlargement. Clear isosbestic points were observed throughout the irradiation at 1636 (± 2) cm^−1^ and 1661 (± 2) cm^−1^ (Figure S1, Supp. inf.). The area of the Amide I band remained constant, even at the highest doses. Gel irradiation experiments with FTIR analyses showed excellent reproducibility of the secondary structure evolution, with an experimental uncertainty of a few percent.

**Figure 1:**
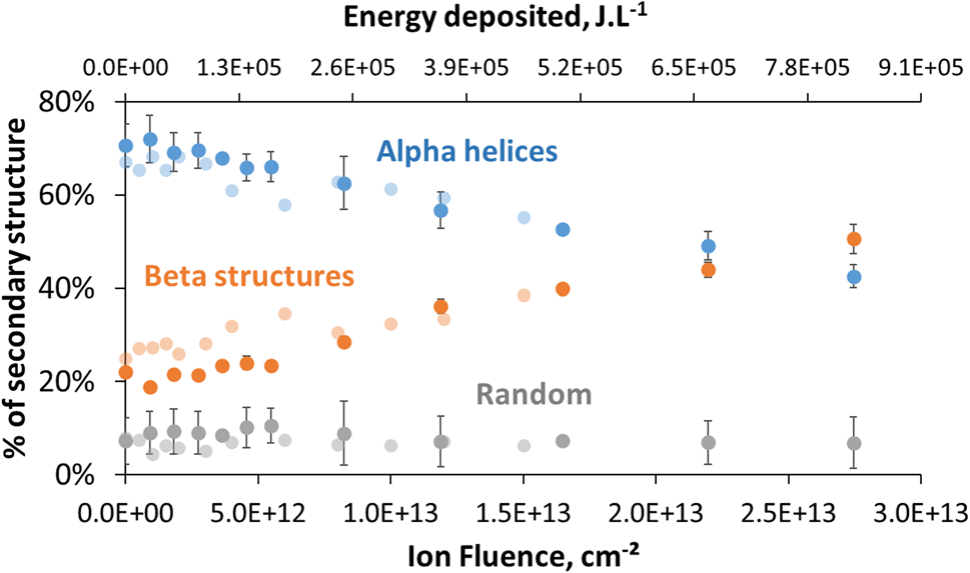
Evolution of the secondary structure of myoglobin under irradiation by 2 MeV protons, as determined by infrared spectroscopy. Light-color circles, 30% metMb solution in D_2_O. Deep-color circles, protein gel, [metMb]_0_ = 1.1 × 10^–2^ M in D_2_O, Gel thickness 26 µm. Error bars were determined with a duplicate of irradiation experiments.

Figure 1 presents the evolution of proportions of the alpha, beta and random structures as a function of ion fluence and energy deposited, calculated with protons average LET. No remarkable change in the secondary structure is observed for fluences below ~ 3 × 10^12^ ions cm^−2^. Above that fluence, irradiation resulted in a decrease of the percentage of alpha helices, almost linear with fluence, from 70 to 43%, to the benefit of beta structures. The proportion of disordered, random, structures remained unchanged throughout the fluence range, around 10%. In order to assess a potential incidence of glutaraldehyde on the variations observed, a concentrated solution of protein was also irradiated, quickly enough to avoid flowing during the experiments. The dataset recorded under irradiation displays very similar evolution of the secondary structure of the protein in solution, compared to the gels (Fig. 1, light symbols). This shows that the cross-linker did not influence the conformation changes of the protein under irradiation.

### FTIR spectra, other bands of interest

**Figure 2:**
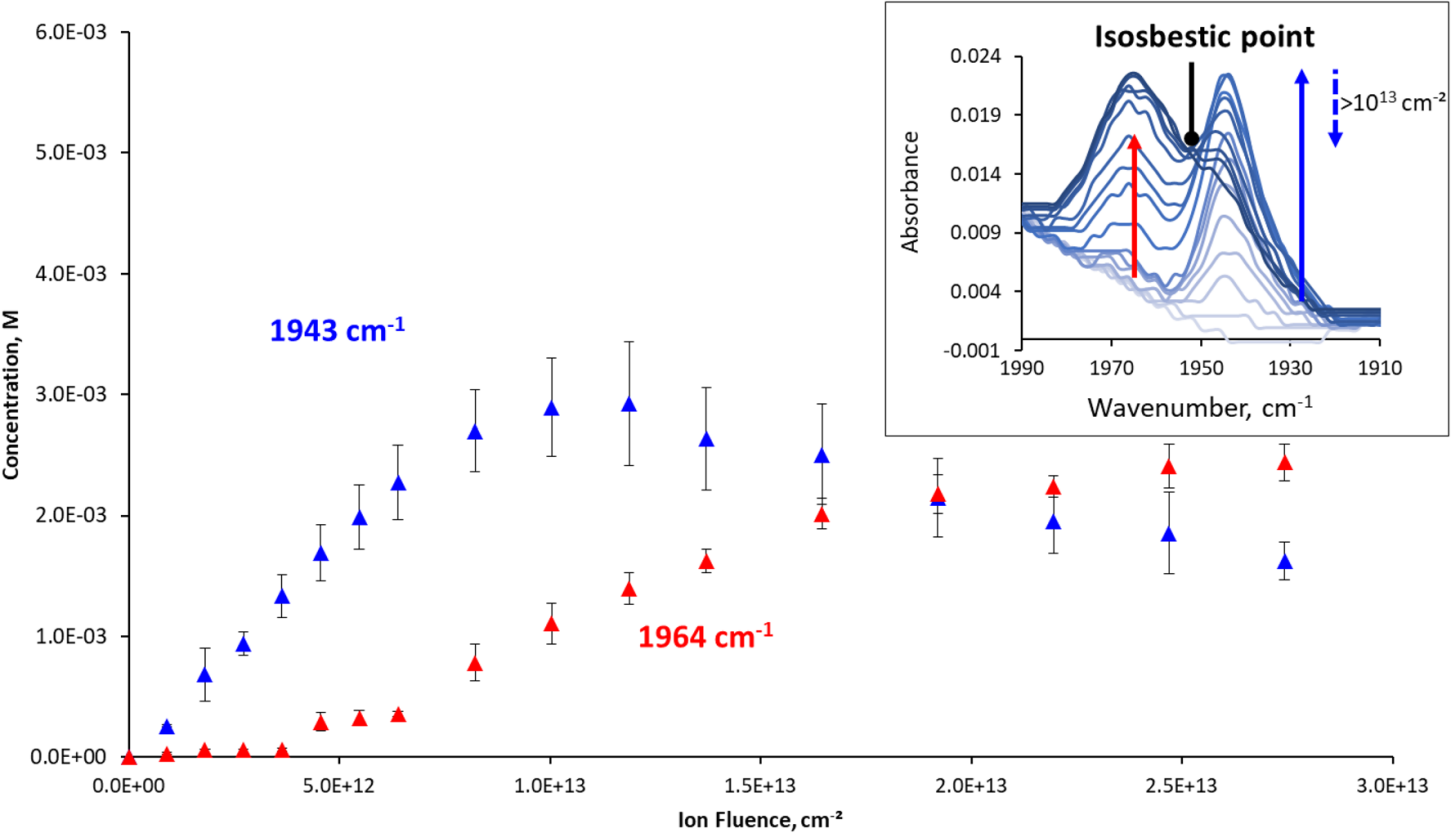
Evolution of the concentrations of carbon monoxide bound to iron complexes with ion fluence. The quantities were determined from infrared measurements of the bands at 1943 cm^−1^ and 1964 cm^−1^. Insert: evolution of IR spectra with increasing irradiation doses, as shown by the arrows. The isosbestic point is observed for ion fluences above 10^13^ ions/cm^−2^. Error bars were determined with a duplicate of irradiation experiments.

Irradiation of myoglobin hydrogels led to the appearance of two absorption bands in the range 1900–2000 cm^−1^ (Fig. 2, insert). The intensities of these signals are very low, however well measurable, thanks to the on-line analysis and reproducibility of the positioning of the sample between each irradiation. At low ion fluence, a band appears and rises at 1943 cm^−1^, with a shoulder around 1933 cm^−1^. This signal is characteristic of carbon monoxide CO bond vibration, when the molecule is bound to a heme^26,27^.

The concentration of the corresponding species was calculated after determining the absorption coefficient of the band at 1943 cm^−1^. To do so, concentrated solution of native myoglobin was first reduced with sodium dithionite, then subjected to carbon monoxide gas and analysed in the same conditions as the gels by infrared and UV–Visible. We were able to estimate the IR molar absorption coefficient at 1943 cm^−1^ of MbCO, by measuring simultaneously infrared and UV–Visible spectra of concentrated solutions, and using the UV–Visible absorption coefficient at 421 nm (see below):

$${\varepsilon }_{{1943 {\text{cm}}}^{-1}}=2991 \pm 370 {M}^{-1}.{{\text{cm}}}^{-1}$$

The concentration of the species absorbing at 1943 cm^−1^ was then determined from absorbance measured (Fig. 2). It increases with ion fluence until reaching a maximum at about 10^13^ ions cm^−2^ and then decreases under further irradiation, in a bell-shaped curve. Above a fluence of 4.5 × 10^12^ ions cm^−2^, another band appears, at 1964 cm^−1^, whose intensity increases regularly with ion fluence. Concentrations of the corresponding species were calculated assuming that ${\varepsilon }_{{1964 {\text{cm}}}^{-1}}$ would be close to ${\varepsilon }_{{1943 {\text{cm}}}^{-1}}$. Absorbance signal at 1964 cm^−1^ has also been attributed to CO bound to a heme, generally associated with less steric hindrance around carbon monoxide^28,29^. A clear isosbestic point can be observed between the two bands for ion fluences above 10^13^ ions cm^−2^ (Fig. 2, insert). This indicates that the first species (1943 cm^−1^) most likely transforms into the one absorbing at 1964 cm^−1^.

### UV–visible analysis

**Figure 3:**
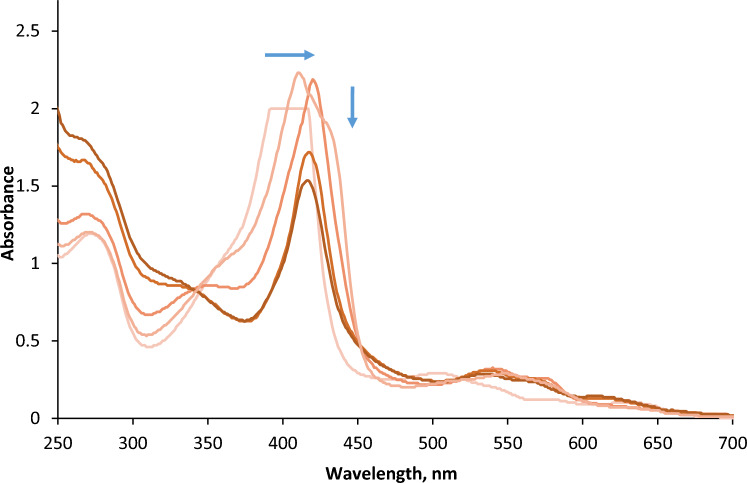
UV–Visible spectra of myoglobin gel recorded for significant ion fluences. The arrows show the evolution of the Soret band with increasing ion fluence up to 2.7 × 10^13^ ions cm^−2^. Soret band of unirradiated protein is saturated due its strong absorption explaining this plateau shape at an absorbance of 2. [metMb]_0_ = 1.1 × 10^–2^ M, in D_2_O. Gel thickness 26 µm.

Combined with infrared spectra, recording of UV–Visible spectra before irradiation allowed characterisation of the gel and determination of its thickness. Spectra were then recorded after successive proton irradiations. The only absorbing species in the range 350–800 nm is the heme of the protein. Upon irradiation, the UV–Visible spectrum showed drastic changes, with a shift of the intense heme Soret band from 408 to 422 nm, combined with the appearance of Q bands at 541 and 578 nm (Fig. 3).

These features are in agreement with the formation of carboxymyoglobin (MbCO) in the irradiated samples, which shows similar characteristic bands^30^. Since metMb needs first to be reduced to fix carbon monoxide, the formation under irradiation of deoxymyoglobin (deoxyMb) was postulated. Radiolysis of myoglobin has been shown to lead to its reduction in several works, in aerated or deaerated medium^19,31–33^. In order to determine the concentrations of the different heme species involved, multicomponent analysis (MCA) was performed on the spectra measured, using a software developed by Gonen and Rytwo^34^, with absorption coefficients determined for the three forms of myoglobin (Table 1).

**Table 1:** Molar absorption coefficients determined for the metmyoglobin, deoxymyoglobin and carboxymyoglobin, at the Soret and Q bands wavelengths.

Globin	ε_Soret_, M^−1^ cm^−1^ (Wavelength)	ε_α_, M^−1^ cm^−1^ (Wavelength)	ε_β_, M^−1^ cm^−1^ (Wavelength)
Metmyoglobin (metMb)	164 × 10^3^ (408 nm)	9.4 × 10^3^ (502 nm)	6.2 × 10^3^ (540 nm)
Deoxymyoglobin (deoxyMb)	121 × 10^3^ (433 nm)^35^	12.6 × 10^3^ (560 nm)	–
Carboxymyoglobin (MbCO)	169 × 10^3^ (421 nm)	14 × 10^3^ (540 nm)	12 × 10^3^ (576 nm)

**Figure 4:**
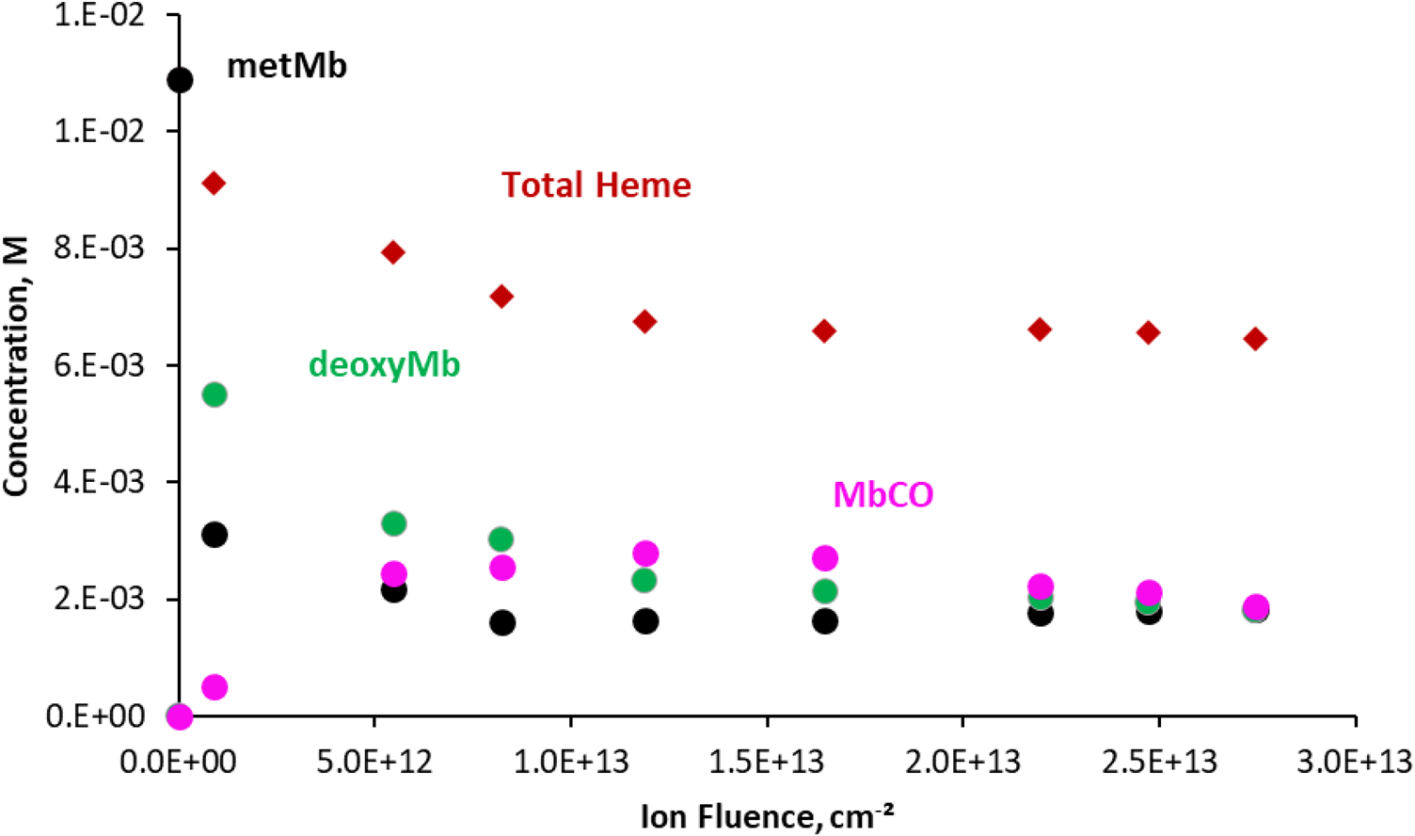
Concentrations of metMb, deoxyMb, MbCO and total heme determined by MCA of the UV–Visible spectra, as a function of ion fluence. Met-Myoglobin, black dots; deoxy myoglobin, green dots; carboxymyoglobin, blue dots; total heme concentration, red diamonds. [metMb]_0_ = 1.1 × 10^–2^ M, in D_2_O. Gel thickness 26 µm.

Figure 4 shows the results obtained with MCA, with the concentrations of the three species, and of the total heme, as a function of the protons fluence. The concentrations of all species were calculated using the molar absorption coefficients and the thickness of the gel determined. The total heme concentration represents the sum of concentrations of MetMb, deoxyMb and MbCO. The quantity of metMb decreases of more than two third following the first irradiation dose, and seems to reach a plateau above 8.2 × 10^12^ ions cm^−2^.

After the first irradiation, deoxyMb, which was not present before irradiation, becomes the main species in the gel. Its quantity decreases with ion fluence, while MbCO quantity rises with fluence up to 1.2 × 10^13^ ions cm^−2^, before slowly decreasing. These observations confirm that the protein is efficiently reduced under irradiation, and that carbon monoxide forms under irradiation, and is fixed by the reduced heme. The total heme concentration decreases with dose up to the same fluence of 1.2 × 10^13^ ions cm^−2^, indicating that its degradation goes along with the formation of deoxyMb and MbCO species.

## Discussion

The results show that the protein undergoes important secondary structure modification under irradiation by low-energy protons and alteration of its heme prosthetic group, with significant formation of CO bound to the heme.

Under irradiation, water radiolysis will lead to formation of several reactive species, which will react with the biomolecule. The secondary structure of myoglobin changes drastically, with sharp decrease of alpha helices proportions, together with an increase in global beta structures, but no noticeable modification of the proportions of random structure (Fig. 1). In all experiments performed, the changes are very similar and reproducible, and lead to very close final secondary structures at high dose. This shows that the modifications observed are not a random degradation of the protein, but actually a conversion of the alpha helices to beta structures, leading to the formation of a denatured protein with a structure consisting of 41% ± 3% alpha helices, 48% ± 4% beta and 11% ± 6% random coils at highest fluence. Puchala and Schueller^23^ have also observed non-random degradation of myoglobin, with specific chain scissions, under gamma rays radiolysis of diluted solutions. Although here we have no evidence for chain scissions, specific damage positions on the protein would explain why the conformation changes lead to a reproducible well-defined structure. The high content of beta structures raises the question of the possible formation of amyloid fibrils by aggregation. Indeed, apomyoglobin has been shown to form such fibrils under specific denaturing conditions^36,37^. Amyloid fibrils are characterized by intermolecular interaction between β-sheets, “cross-β” structures^38^. Here, FTIR spectra of the irradiated samples showed no significant peak in the region 1615–1620 cm^−1^, which would be characteristic of these cross-β structures^37,39,40^ This would indicate that no amyloid fibrils form in our samples. After irradiation, even at low fluences, protein gels become completely insoluble, whereas the gels of non-irradiated protein can be dissolved in sodium dodecylsulfate solutions. This shows that covalent reticulation occurs between the proteins, in addition to the conformation changes observed. Such aggregation by cross-linking under irradiation is quite common, and has been observed with X-rays, UV or γ-rays^8,41,42^, although chain-scission can be predominant, depending on the conditions^12^. Such rapid cross-linking in the samples could prevent more global inter-protein rearrangements of the proteins, which can lead to formation of beta fibrils. So, although no cross-β structures were observed during the experiments, it cannot be completely discounted that they could form through irradiation at lower concentrations, where covalent cross-linking becomes less favourable.

Both infrared and UV–Visible analyses are in agreement with the formation of heme-CO complex under irradiation. MCA decomposition of the UV–Visible spectra showed how individual concentrations of metMb, MbCO and deoxyMb evolve gradually with dose. It should be noted that UV–Visible spectra do not indicate whether the observed heme is associated with the native or denatured protein. As for formation of deoxyMb, reduction of heme ferric iron to ferrous iron was already observed under pulse radiolysis ferricytochrome C or methemoglobin with accelerated electrons^33,43^. The reaction of hydrated electron, produced by water radiolysis, was actually found to proceed at almost diffusion-controlled rate. The heme acts as an effective electrophilic sink for e^−^_aq_^44^, despite the fact that it is buried in the protein^45,46^. Binding of e^−^_aq_ to the protein polypeptide chain followed by rapid transfer to the heme is also a possible mechanism. Carbonyl groups on the surface have been suggested as initial sites of electron attachment^47^. H^•^, which is expected to be produced in lower quantity than e^−^_aq_, also reacts with the protein and can lead to reduction of the heme. HO radical has also been described to lead to heme reduction, through formation of radicals after reaction with side-chains of protein residues^19,33^. In this work, the high density in protein in the gel favours the reaction of the heme with species produced by water radiolysis before their scavenging by molecular oxygen.

After the first irradiation fluence with low-energy protons, most of the Iron(III) centre in the heme has been reduced to Iron(II) (Fig. 4), and can therefore fix carbon monoxide molecules. Indeed, MbCO concentration determined by UV–Visible spectroscopy increases and it becomes the most important species above a fluence of 1 × 10^13^ ions cm^−2^. Capture of carbon monoxide by myoglobin has been widely studied, since poisoning by this gas makes victims every year. Carbon monoxide is also a good probe to study the heme binding site by vibrational spectroscopy^48^. As soon as CO is produced, it is expected to be trapped by the protein, since the affinity of the free heme ferrous iron for CO is 200 times greater than for O_2_^49^. Therefore, one can expect most of the CO formed to have been trapped by the reduced heme.

**Figure 5:**
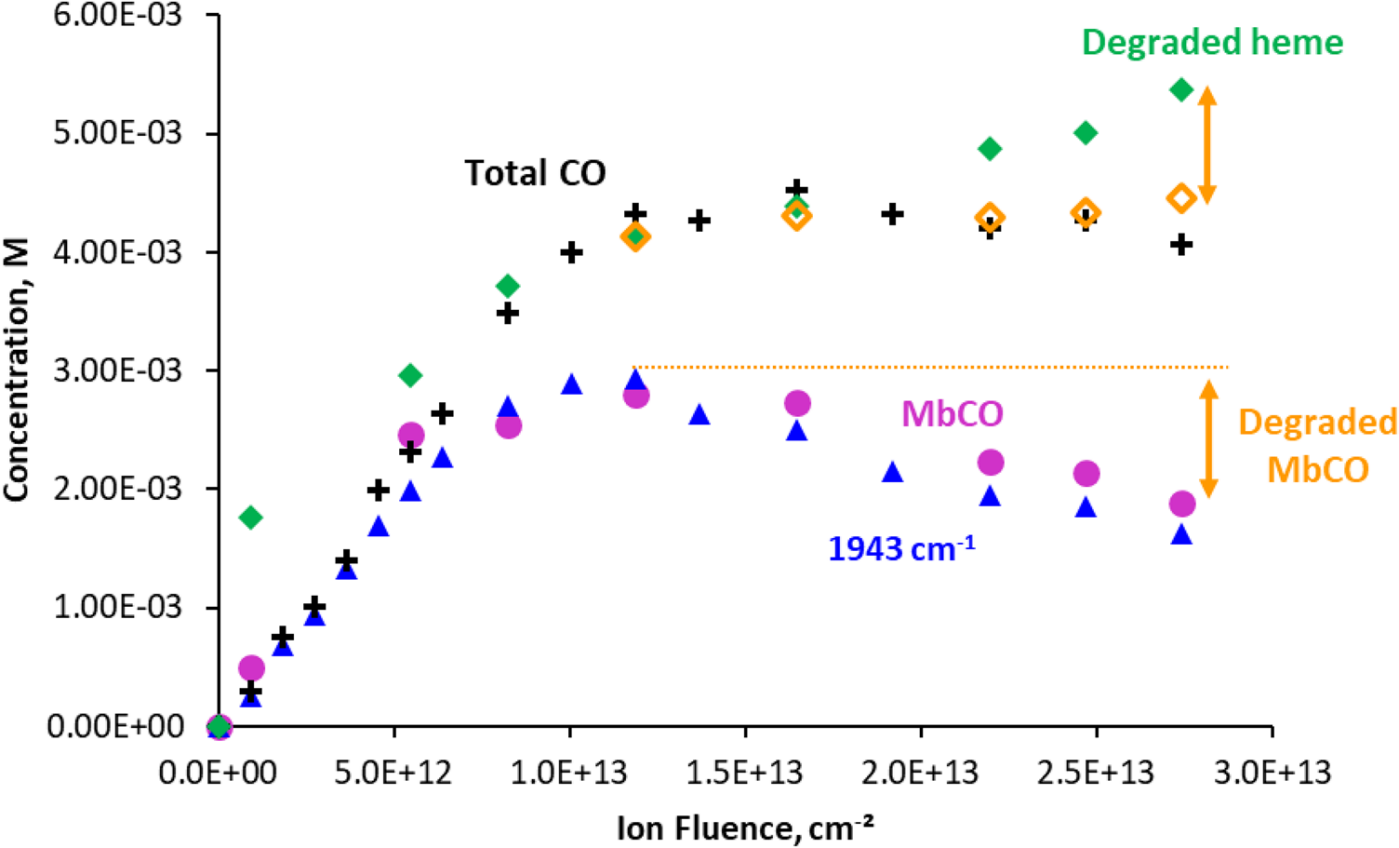
Comparison between the evolutions of quantities of carboxymyoglobin MbCO, determined by UV–Visible spectrometry with that of the species absorbing at 1943 cm^−1^ in infrared, and of total CO measured and global heme degradation, under irradiation. +: total CO concentration, blue triangle: concentration of the species absorbing at 1943 cm^−1^, pink circle: MbCO concentration, green diamond: global concentration of degraded heme, Yellow open diamonds: Global quantities of degraded heme after subtraction of MbCO degraded.

When bound to Mb heme, the CO vibrational frequency is known to be substantially red-shifted from the gas-phase frequency and separated into several distinct bands, peaking at 1966, 1945 and 1933 cm^−1^ on FTIR spectra. These bands could be attributed to three different conformational substrates, denoted as A0 (1966 cm^−1^), A1 (1945 cm^−1^), and A3 (1933 cm^−1^)^50^. A0, A1 and A3 differ in the orientation of the CO dipole with the heme^51,52^, which is controlled by the distal histidine (His64)—CO interactions^53^. A first hypothesis would be that the bands at 1943 cm^−1^ and 1964 cm^−1^ could correspond to A1 and A0 forms respectively. The species responsible for the vibration at 1964 cm^−1^ could also be a heme with modified polar interactions with distal residues^48^, or a species derived from a heme, such as a chlorin^54^ or α-hydroxyheme^55^. To test these hypotheses, the evolutions under irradiation of the concentrations of various species identified were compared. If the species absorbing at 1964 cm^−1^ corresponded to A0 less constrained form, one would expect a strong correlation between its formation and the evolution of the secondary structure of the protein. Here, when its formation was compared to that of denatured protein, no correlation could be observed between the two species. Figure 5 shows that the concentrations of species absorbing in IR at 1943 cm^−1^ are identical to that of MbCO determined by UV–Visible spectroscopy, having the exact same evolution with ion fluence. This means that the CO absorbing at 1943 cm^−1^ corresponds to MbCO formed in its entirety. Therefore, the CO species absorbing at 1964 cm^−1^ must have UV–Visible absorption spectra significantly different from ferrous heme-CO complex, and is not observed with UV–Visible spectroscopy. As a result, this species is most likely not a less constrained heme A0, but is rather derived from heme degradation.

In order to determine the source of CO observed under irradiation, we performed two control experiments (Data not shown). When a concentrated solution of myoglobin was irradiated without addition of glutaraldehyde, carbon monoxide was still observed, which shows that the crosslinker is not the main source of CO, if it produces any. Then, polypropylene films with only deuterated water in between were irradiated. These were directly linked to a gel of deoxyMb formed by reaction with sodium dithionite, which was not irradiated, and served as a probe for CO formation. The UV–Visible absorbance of the deoxyMb gel was measured after each irradiation, and showed no change in the Soret band, indicating that no carbon monoxide was formed by the polyethylene films. So, the source of carbon monoxide produced under irradiation is definitely the protein.

There are a few reports on the formation of CO under irradiation under kGy irradiation of polysaccharides by gamma rays^56^, of amino acids, phospholipids and some proteins by fast electrons, with little data on the mechanisms involved^57^. Carbon monoxide is also naturally produced in organisms, as an elimination product of metabolism. The main mechanism of biosynthesis is by heme degradation, catalysed by heme oxygenases^58^. The first step is the addition of a hydroxyl group on the α-methine bridge of the heme, followed by several oxidation and reduction steps, leading ultimately to the formation of CO, α-bilirubin and free iron(III)^59^. There could be several pathways for the heme degradation, with O_2_ and reducing agents, O_2_ alone^60^, or hydrogen peroxide^61^.

The total heme content, determined as the sum of metMb, deoxyMb and MbCO concentrations, decreases with dose, indicating that it is degraded under irradiation (Fig. 4). The quantity of degraded heme is calculated as the difference between initial heme concentration and that of metMb, deoxyMb and MbCO. Figure 5 shows that the quantities of degraded heme follow quite closely that of total carbon monoxide detected with IR bands at 1943 cm^−1^ and 1964 cm^−1^. Horejsi et al*.* have shown that addition of 7,8-dihydroneopterin to myoglobin or haemoglobin leads to complete degradation of the heme and equimolar production of carbon monoxide. 7,8-dihydroneopterin is known to produce hydroxyl and superoxide radicals in air-saturated solutions^63^, which could lead to a degradation similar to that observed in this work. It appears from these observations that the heme itself is most likely the main source of the carbon monoxide observed under proton irradiation, probably through reactions involving hydrogen peroxide, hydrated electron and hydroxyl radical produced under irradiation, and O_2_ dissolved in solution. This also supports the proposition that the IR band at 1964 cm^−1^ could be ascribed to carbon monoxide linked to a species formed by degradation of the heme, with quite different UV–Vis spectra. At high ion fluence, a deviation can be observed between the global quantities of heme degraded and that of CO formed. While the first one increases with fluence, total CO concentration remains stable (Fig. 5). As mentioned above, the quantity of MbCO evolves as a bell-shaped curve, reaching a maximum at a fluence of about 10^13^ ions cm^−2^, followed by a decrease indicating the degradation of the species (Fig. 2). When the concentration of MbCO degraded is subtracted from the total heme degraded, this last falls back exactly to the amount of CO formed, as shown on Fig. 5. It can be concluded from this observation that the destruction of MbCO does not lead to additional formation of carbon monoxide. Since total carbon monoxide quantity remains stable above 10^13^ ions cm^−2^, this means that above this fluence, we see either only a transfer of CO from degraded MbCO to the degraded heme absorbing at 1964 cm^−1^, or direct conversion of MbCO into the species absorbing at 1964 cm^−1^. This is in line with the isosbestic point observed above this fluence between the 1943 and the 1964 cm^−1^ bands in the infrared spectra, indicating an interconversion of the two species (Fig. 2, insert).

**Figure 6:**
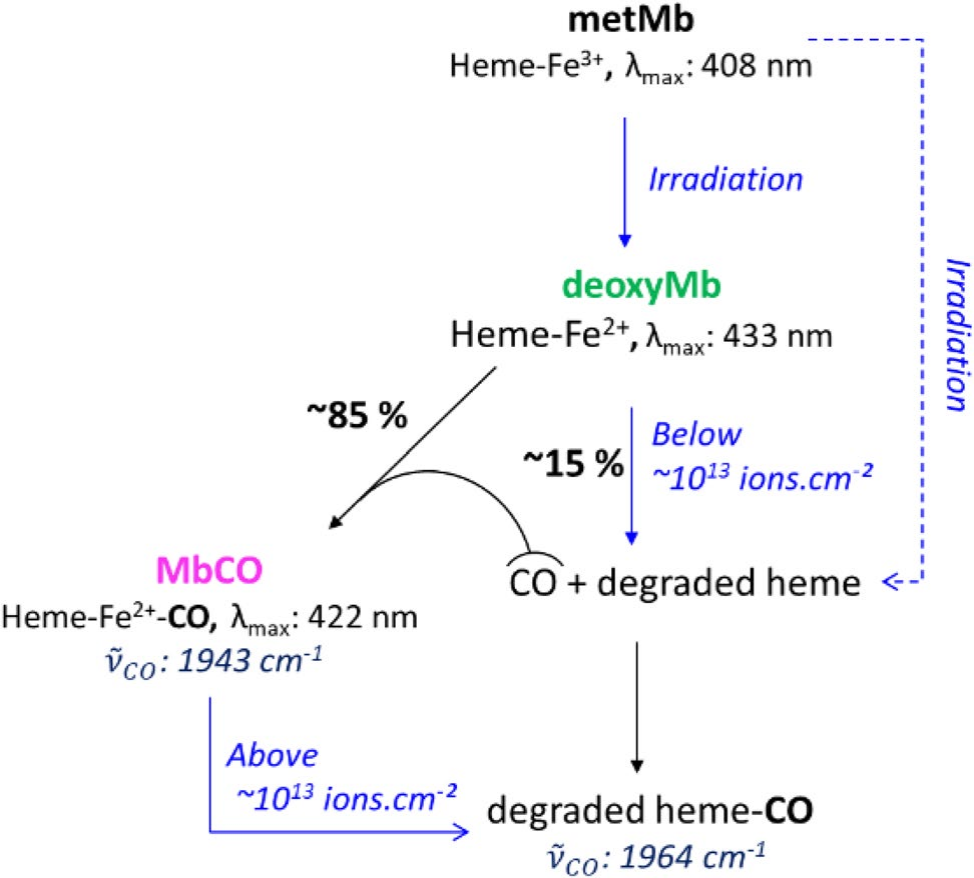
Proposed description of the formation of heme species identified in myoglobin radiolysis.

Figure 6 summarizes the conclusions drawn from the above discussion. In the first steps of irradiation, metMb is reduced to deoxyMb and eventually also directly degraded. Heme degradation, either from deoxyMb or metMb, leads to the formation of CO, which is fixed by deoxyMb to yield carboxymyoglobin MbCO, and to some extent by the degraded heme, leading to IR absorption at 1964 cm^−1^. Above a fluence of about 10^13^ ions cm^−2^, no additional formation of CO or heme degradation is observed, except for that of MbCO. So, there must be a competition under irradiation between the degradation of deoxyMb and that of MbCO, strongly in favor of the latter above 10^13^ ions cm^−2^, when it becomes the most abundant heme species. Degradation of MbCO does not yield CO, as shown above, which explains why no additional carbon monoxide is formed under irradiation above 10^13^ ions cm^−2^. Heme degradation leading to CO formation is known to be inhibited by CO itself, especially preventing the conversion of verdoheme to biliverdin^64^. While heme oxygenases have structural features that prevent such inhibition, under irradiation, carbon monoxide most likely inhibits efficiently the degradation.

Experiments in this work were performed under aerated conditions, with most water radiolysis species reacting with the protein and the heme. Studies with gamma-rays have shown that evolution of myoglobin protein and its heme depends on O_2_ content^21,23^. O_2_ is expected to play a role in the degradation of the protein described here, either by reacting with carbon-centred radicals formed after reaction with HO^•^, to yield peroxyl radicals, or in the heme degradation leading to formation of carbon monoxide. At this stage, the precise mechanisms of degradation cannot be described, and will require dedicated studies with controlled oxygen concentration and specific HO^•^ and e^−^_aq_ scavengers.

Beyond mechanistic considerations, we sought to quantify the species formed by radiolysis as a function of the energy deposited, i.e. to determine their radiolytic yields. Radiolytic yield G_±_ represents the number of any species formed (G_+_) or consumed (G_−_) per energy unit deposited by an ionizing radiation, either in mol/J or species/100 eV. It is commonly used to quantify and compare the amounts of species formed or degraded per unit of energy under irradiation, and is therefore useful for comparison between various irradiation conditions (type of ionizing radiation, energy etc.). Here, it could be estimated as the slope of the number of moles per Joule deposited.

At 2 MeV energy, the average LET of protons in a 26 µm gel is 17.7 ± 1 eV/nm, determined by SRIM software^65^. Therefore, the thickness of the gel being precisely determined, the energy deposited in J per litre in the gel was computed from the fluence. Quantities of total CO, MbCO and degraded heme were determined as described above. As for the intact Mb and the denatured protein, their quantities were determined by deconvolution of average secondary structure. This average structure is considered a linear combination of both structures, determined respectively before irradiation and at maximum fluence.

**Figure 7:**
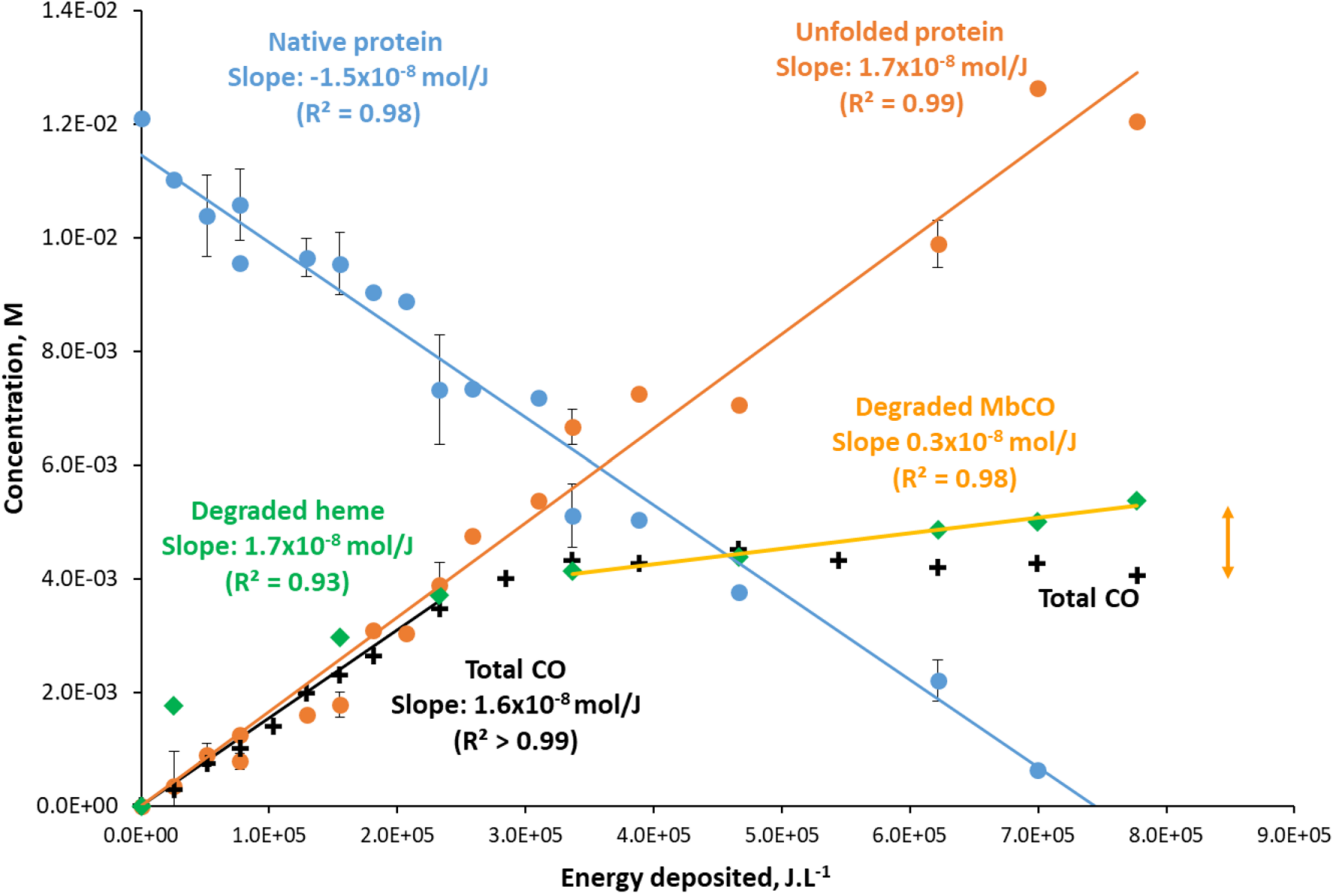
Evolution of the concentrations of native myoglobin (blue dots), denatured protein (orange dots), of degraded heme (green diamonds) and total CO (black crosses), with the energy deposited in the sample per liter. Error bars on native and denatured protein concentrations were determined with a duplicate of irradiation experiments on two gels.

Figure 7 shows the evolution of the concentrations of native and denatured proteins as a function of the energy deposited by protons. These quantities evolve linearly with the energy deposited, showing a clear dose–effect on the conversion of the native protein under irradiation.

The quantities of degraded heme and of total CO formed are also represented and follow quite closely that of denatured protein, up to an energy deposited of 3.2 × 10^5^ J L^−1^, which corresponds to an ion fluence of 10^13^ ions cm^−2^. This close correlation between degradation of the heme, production of CO, and unfolding of the protein, which occur with almost 1:1 ratio, could mean that when an accelerated proton interacts with the protein, the energy deposited causes simultaneously the unfolding of the biomolecule, and the degradation of the heme. A cooperative process could be considered, in which residues of the denatured protein could be involved in the heme degradation mechanism. The observation that heme degradation and CO production stop beyond 3.2 × 10^5^ J L^−1^, while the unfolding of the protein steadily continues, can be attributed to a competition favoring MbCO degradation at high fluence, as discussed above. The radiolytic yields of the four species could be calculated from the slopes of the curves of Fig. 7. They were found very close, within the limits of uncertainty:

$$G_{{ + {\text{denatured Mb}}}} \approx \, G_{{ + {\text{CO}}}} \approx \, G_{{ - {\text{heme}}}} = \, 1.6 \times 10^{ - 8} \pm \, 0.1 \times 10^{ - 8} \;{\text{mol}}/{\text{J}} = \, 0.16 \, \pm \, 0.01{\text{ species}}/100\;{\text{eV}}$$

The rise of degraded heme compared to that of total CO above 3.2 × 10^5^ J L^−1^, corresponds to the degradation of MbCO. The radiolytic yield of degradation of MbCO could be estimated using the slope of the curve of heme degradation beyond 3.2 × 10^5^ J L^−1^ deposited (Fig. 7, yellow line):

$${\text{G}}_{{ - {\text{MbCO}}}} = \, 0.{3} \times {1}0^{{ - {8}}} \;{\text{mol}}/{\text{J}} = 0.0{3}\;{\text{species}}/{1}00\;{\text{eV}}$$

This yield is more than five times lower than the degradation of heme under irradiation, confirming that the fixation of carbon monoxide on the heme iron reduces its degradation.

At concentration levels close to that encountered in living cells, radiolysis of myoglobin by Bragg-peak energy protons leads to major changes in the secondary structure of the protein, with a conversion of alpha helices to beta structures. No cross-β structure was detected, which may be related to the covalent cross-linking that occurs under irradiation. Changes in the secondary structure of the protein are associated with loss of biological activity, and may lead to new activities. Here, they are accompanied by a significant production of carbon monoxide, from the degradation of the heme. Further studies will be needed to better describe the radiolysis mechanisms leading to the conformational changes of the protein, and the formation of CO. This last could play a critical role in the toxicity of the irradiation, as carbon monoxide will have an adverse effect on the production of energy by the cell^66^. At low concentration, it can modify the mitochondrial respiration and increase the production of ROS^67^. Cancer cells having a high metabolism, they will be especially sensitive to CO, which has been considered as a potential therapeutic agent^68^. Moreover, carbon monoxide is a signalling molecule and can activate intracellular ion channels^69^, pass through biological membranes^70^, and could therefore have an effect also on the neighbouring cells. This could play a significant role in the bystander effect, whereby non-irradiated cells surrounding an irradiated cell die.

## Methods

### Chemicals

Horse heart Myoglobin (Met-Myoglobin, metMb) was purchased from Sigma-Aldrich (purity ≥ 90%). It was purified before use, following the procedure described below. Deuterated water (Sigma-Aldrich, 99.9% isotopic purity) and glutaraldehyde (TCI, 50% in water w/w) were kept under nitrogen atmosphere to avoid contamination by water vapor after first opening. Polypropylene sheets (4.75 µm  ±  3%, Spex Sample Prep) and steel foil (5 µm) were used for protein hydrogel preparation and irradiation. Bovine serum albumin (Euromedex, > 98%), Human serum albumin (Sigma-Aldrich), and β-lactoglobulin from bovine milk (Sigma-Aldrich, ≥ 90%, mixture of 2 variants) were used after similar purification to that described for myoglobin.

### Purification and concentration

The lyophilised Met-Myoglobin was dissolved in deuterated water, to reach a concentration of ~ 5% (w/w). 500 µL of the solution were introduced in a short polyethylene tube sealed on one face with a dialysis membrane (Cellulose, Biodesign Dialysis Tubing, Fisher, cutoff 8 kDa). The sample was dialyzed twice against 4.5 mL of deuterated water at 4 °C. D_2_O was renewed after 4 h, to reach a dilution coefficient of 1000 for impurities, and sample was left overnight in D_2_O. After dialysis, the solution was centrifuged 10 min at 9000 rpm in order to eliminate aggregates. The supernatant was concentrated using centricon tubes (3 kDa cut-off) and the concentration was adjusted to obtain a solution with a final protein content of about 25% (w/w). Exact final concentration of the protein was determined from UV–visible measurements.

### Circular dichroism measurements

Lyophilised protein was dissolved in water, to reach a concentration of ~ 5% (w/w). After dialysis at 4 °C (Cellulose, Biodesign Dialysis Tubing, Fisher, cutoff 8 kDa), the solution was placed in a 15 mL tube and centrifuged 10 min at 9000 rpm to eliminate aggregates. The supernatant was then diluted to about 30 mg L^−1^, a concentration suitable for circular dichroism analysis with a 1 mm optical path quartz cell. Spectra were recorded between 185 and 260 nm on a Jasco J-180 spectrometer at a scan speed of 100 nm/min with a resolution of 1 nm and using 6 accumulations. The temperature was maintained at 20 °C during the measurement with a Peltier module. The blank signal, measured with water, was systematically subtracted from the protein signal and a smoothing of 15 points using Savitzky–Golay algorithm was applied. Resulting spectra were deconvoluted on Dichroweb, using the CONTIN algorithm^71^.

### Hydrogels preparation

Protein hydrogels were obtained by reacting highly concentrated protein solutions (25% w/w) with a 2% w/w glutaraldehyde solution (stock commercial 50% solution freshly diluted in D_2_O). Gelation was achieved using a molar glutaraldehyde-to-protein ratio of 3.3, under strong stirring. After mixing of the reactants, 5 µL of the very viscous solution were introduced between two polypropylene films spaced with 5 µm steel foil. The whole sample was pressed under 10 kg for few minutes in order to obtain a homogeneous 10 to 30 µm thick hydrogel expanded over a few square centimetres. The thickness of the sample was determined by combining infrared absorption of deuterated water and UV–visible absorption of the protein.

### Irradiation setup and on-line FTIR and UV–Visible measurements

A lab-made 3D-printed cell was built to allow IR and UV–Visible analyses, on-line with ion irradiation, and with minimal manipulation of the samples (Figure S2, Supp. Inf.). The cell includes two ZnSe windows (Crystran), allowing good transmission of infrared signal between 4000 and 700 cm^−1^ and a support dedicated to the hydrogel, so that the latter is moved with a high reproducibility, suitable for spectroscopy measurement. The gel was sequentially irradiated, analysed within less than one minute by infrared spectroscopy and UV–Visible spectroscopy using optic fibers connected to a diode-array detector (DAD, Ocean Optics). This cycle was repeated for each ion fluence. FTIR spectra were recorded on an Alpha Brucker spectrometer using the software OPUS v10, between 4000 and 700 cm^−1^, averaged over 32 scans, with a resolution of 2 cm^−1^. A reference in air was recorded between each irradiation. Protein gels were contained between two 5 µm polypropylene sheets. Polypropylene was chosen for its transparency in the visible spectrum, and for the absence of overlap of its absorption in the infrared range with the Amide I band of the protein.

The irradiation source was a Van de Graaff particle accelerator (Acacia platform, Icube, Strasbourg), producing a proton beam with an initial energy of 2.5 MeV, corresponding to 2.0 MeV in the gel, according to energy loss calculations using SRIM software (The Stopping and Range of Ions in Matter Software, SRIM-2013, http://www.srim.org^65^). Proton source is continuous, and experiments were carried on with an ion current of 2.4 nA cm^−2^. Ion current was monitored with an internal Faraday cup equipped with an electron repeller, during the irradiations. The beam was scanned, to ensure homogeneous irradiation of the sample, with x/y frequencies of 64 Hz and 517 Hz. Ions were extracted from vacuum through a circular aluminized mylar window (15 mm diameter, 12 µm thick, Goodfellow).

### Determination of the secondary structure with FTIR data and validation of the method

Treatment of the FTIR spectra and band attribution were based on a procedure described by Yang et al.^25^ It consists in a 9-points smoothing and second derivation of the Amide I band, using the Savitzky–Golay algorithm^72^. Baseline was subtracted by using local minimum method. Deconvolution of the resulting spectra was performed with the Fityk software (fityk 0.9.8, https://fityk.nieto.pl^73^). Band attribution was based on data reported in the literature^25^.

**Figure 8:**
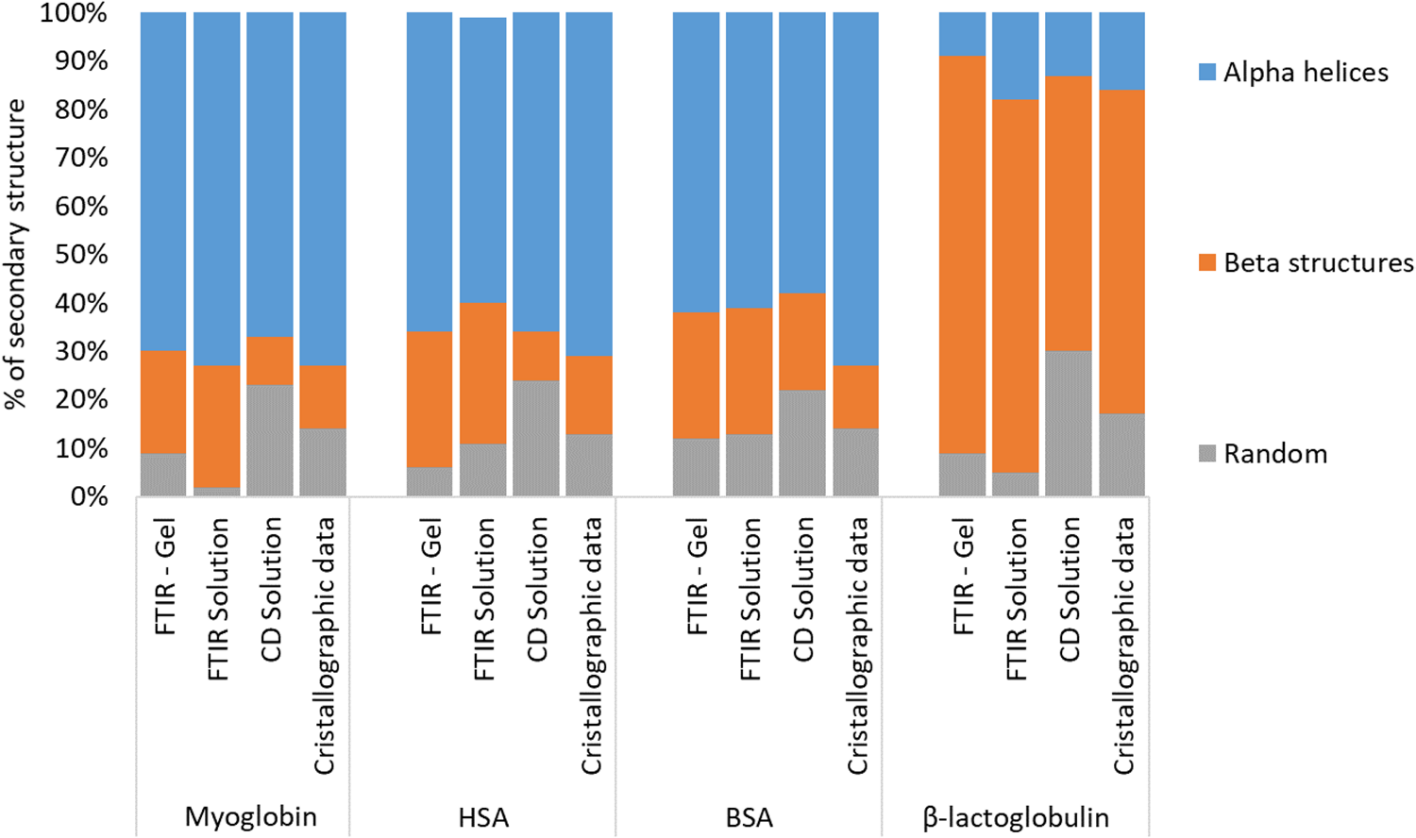
Secondary structures determined by various methods for myoglobin, HSA, BSA and β-lactoglobulin. The crystallographic data used for comparison were determined from pdb structures 1WLA^18^, 1AO6^74^, 3V03^75^ and 1BEB^76^ respectively.

Similar hydrogels were prepared with met-Myoglobin, Bovin and Human Serum albumins (BSA and HSA, resp.) and β-Lactoglobulin, and the secondary structures were determined from FTIR spectra with the same procedures. To estimate the influence of glutaraldehyde on secondary structure, the FTIR spectra of concentrated solutions were measured, without any cross-linker. 2 µM solutions were also analysed by circular dichroism (CD). Figure 8 shows a comparison between the secondary structures determined and crystallographic data from literature. Structures of proteins in gels and in concentrated solutions are very close, within the uncertainties, showing that the cross-linker did not alter significantly the average secondary structure of the macromolecules. Both structures are also quite close to that measured in diluted solutions by CD, and to crystallographic data. This validated the experimental procedure and data processing to determine the secondary structure of proteins under our conditions.

### Determination of UV–Visible molar absorption coefficients

Molar absorption coefficients of the heme in ferric myoglobin (metMb) and ferrous myoglobin linked to carbon monoxide (MbCO) were determined by subjecting four different micromolar solutions (4, 5.8, 7.8 and 9.6 µM) of metMb to the same protocol. In a 1 cm optical path quartz cuvette, 2 mL of metMb solution were introduced, and the UV–Visible spectrum was recorded. An excess of sodium dithionite was then added, to reduce quantitatively heme iron, and the new spectrum was recorded, with Soret and α bands at 433 and 560 nm respectively. These bands are characteristic of the protein with a reduced heme, deoxymyoglobin (deoxyMb). The resulting solution was saturated with CO by bubbling, leading to quantitative formation of MbCO. UV–Visible spectrum was recorded, with characteristic Soret and α, β bands at 422, 543, 578 nm respectively. The absorption coefficients of all three myoglobin forms were determined between 390 and 650 nm, using the absorption coefficient of deoxymyoglobin described by Boutaud et al*.*^35^ (Figure S3 and Table S1, Supp. inf.). Our values for ${\varepsilon }_{Soret}$ are close, but somewhat lower than those reported by Wood^77^ or Harrison and Blout^78^, although with the same ${\varepsilon }_{280 {\text{nm}}}$ than the latter for met-Myoglobin (${\varepsilon }_{280 {\text{nm}}}$ = 34.4 × 10^3^ M^−1^.cm^−1^) (Table S1, Supp. Inf.). ${\varepsilon }_{280 {\text{nm}}}$ could not be determined for deoxyMb and MbCO, due to the strong absorbance at this wavelength of sodium dithionite used for reduction of metMb.

### Determination of infrared molar absorption coefficient at 1943 cm^−1^

metMb in a concentrated D_2_O solution was reduced by addition of an excess of sodium dithionite, and the solution was saturated with CO gas. The solution was placed between two polypropylene films, and UV–Visible and infrared spectra were recorded in the specific cell described above, varying the thickness of the sample. Using the UV–Visible absorbance measured at 422 nm and the molar absorption coefficient ${\varepsilon }_{422 {\text{nm}}}$ of MbCO determined previously, the molar coefficient of the infrared band at 1943 cm^−1^ was estimated with the infrared absorbance measured at this wavenumber.

### Supplementary Information

Supplementary Information.

## Data Availability

Data of metMb MbCO and deoxyMb spectra have been uploaded to figshare. https://figshare.com/s/2dd3543bf8aaafa3b21c.
