# Supplementary material for: Radiolysis of myoglobin concentrated gels by protons: specific changes in secondary structure and production of carbon monoxide
Source: Sci Rep. 2024 Apr 14;14:8625. doi: 10.1038/s41598-024-58378-z (PMC11016545; doi:10.1038/s41598-024-58378-z)
Supplement: Supplementary file 1 — Supplementary Information. [file 41598_2024_58378_MOESM1_ESM.docx]

**Authors**

Nicolas Ludwig^1,2^, Catherine Galindo^1^, Clea Witjaksono^1,3^, Antoine Danvin^1^, Philippe Peaupardin^1^, Dominique Muller^4^, Tamon Kusumoto^5^, Satoshi Kodaira^5^, Rémi Barillon^1^ & Quentin Raffy^1^*

^1^IPHC, Université de Strasbourg and CNRS, UMR 7178, 23 rue du Loess, F-67037 Strasbourg, France

^2^Aerial, Parc D’innovation, 250 Rue Laurent Fries, F-67400, Illkirch, France

^3^Institut de Chimie, Université de Strasbourg and CNRS, UMR 7177, 4 rue Blaise Pascal, F-67070 Strasbourg, France

^4^ICube, Université de Strasbourg and CNRS, UMR7357, 23 rue du Loess, F-67037 Strasbourg, France

^5^National Institutes for Quantum and Radiological Science and Technology (QST), 4-9-1 Anagawa, Inage-ku, 263-8555 Chiba, Japan

Supplementary information


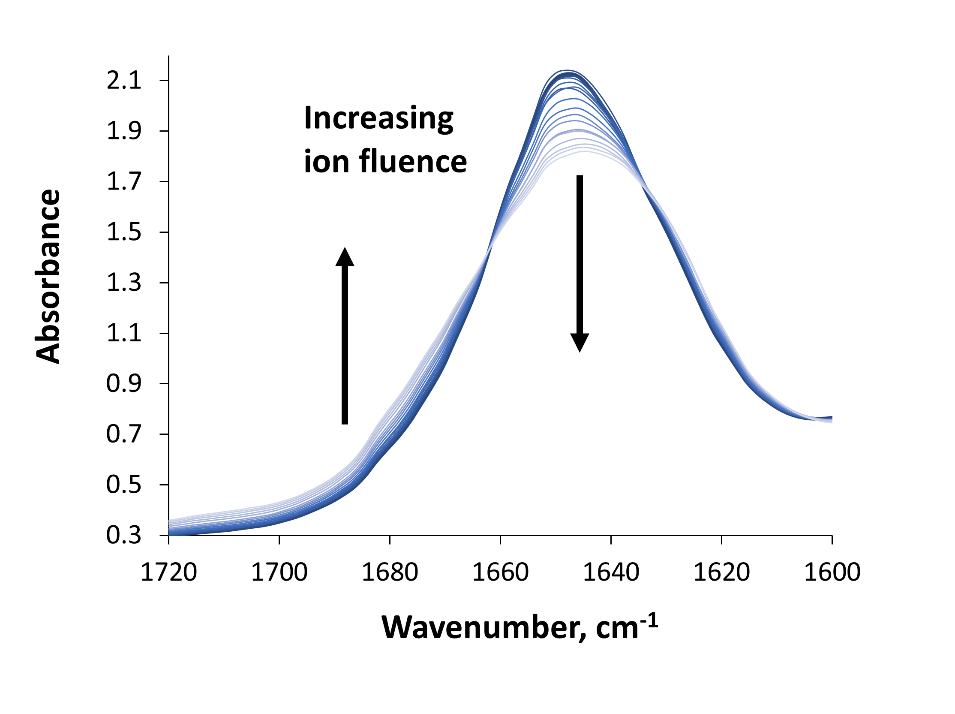


Figure S1. Evolution of amide I band of myoglobin with protons fluence. *
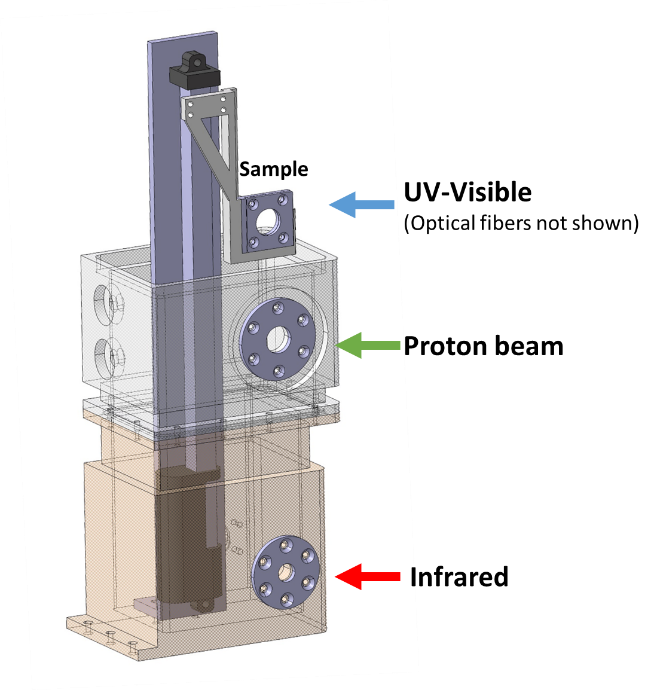
*

Figure S2. Scheme of the 3D-printed cell used for experiments. The sample is placed on an actuator and successively moved between three positions: middle position for protons irradiation, then bottom position for measurement of infrared spectra, and highest position for measurement of UV-Visible spectra. Recording of spectra was done between 30 seconds and 1 minute after each irradiation.

*
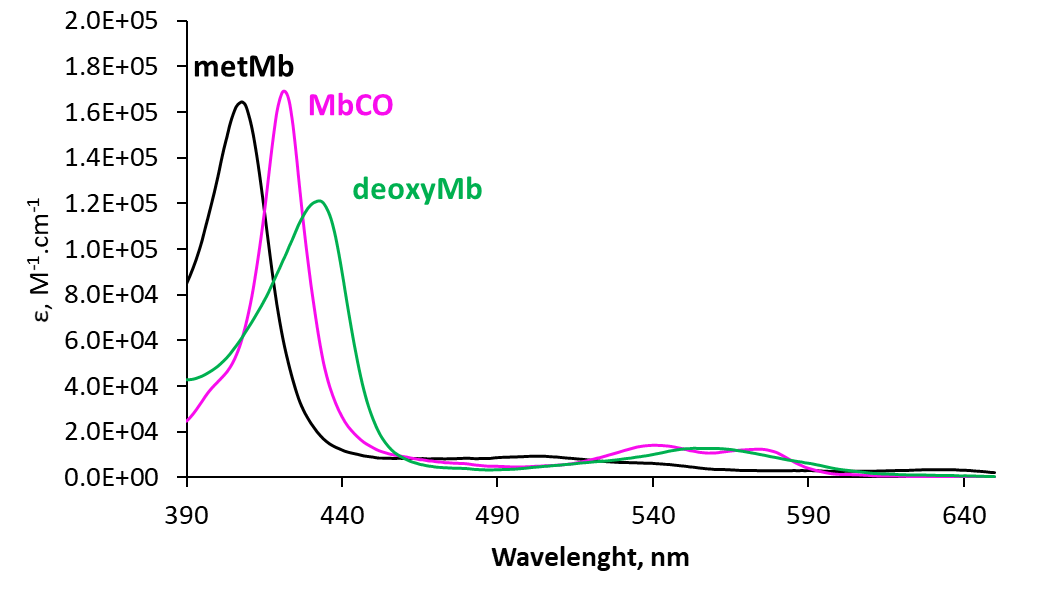
*

Figure S3. Molar absorption coefficients measured for met-myoglobin (metMb), myoglobin linked to carbon monoxide (MbCO) and deoxymyoglobin (deoxyMb).

Table S1: Molar absorption coefficients determined for the met-Myoglobin, deoxy-Myoglobin and carboxymyoglobin, at the Soret and Q bands wavelengths. ^a^ This study

| **Globin** | **ε_Soret_, M^-1^.cm^-1^ (Wavelength)** | **ε_α_, M^-1^.cm^-1^ (Wavelength)** | **ε_β_, M^-1^.cm^-1^ (Wavelength)** |
| --- | --- | --- | --- |
| Metmyoglobin (metMb) | 164x10^3^ (408 nm)^a^  179x10^3^ (408 nm)^26^  188x10^3^ (408 nm)^25^ | 9.4x10^3^ (502 nm)^a^  10.2x10^3^ (502 nm)^25^ | 6.2.10^3^ (540 nm)^a^ |
| Deoxymyoglobin (deoxyMb) | 121x10^3^ (433 nm)^24^  121x10^3^ (435 nm)^25^ | 12.6x10^3^ (560 nm)^a^  13.0x10^3^ (560 nm)^25^ | / |
| Carboxymyoglobin (MbCO) | 169x10^3^ (421 nm)^a^  207x10^3^ (424 nm)^25^ | 14x10^3^ (540 nm)^a^  15.4x10^3^ (540 nm)^25^ | 12x10^3^(576 nm)^a^  13.6x10^3^(579nm)^25^ |
